# Post-Traumatic Stress among Evacuees from the 2016 Fort McMurray Wildfires: Exploration of Psychological and Sleep Symptoms Three Months after the Evacuation

**DOI:** 10.3390/ijerph16091604

**Published:** 2019-05-08

**Authors:** Genevieve Belleville, Marie-Christine Ouellet, Charles M. Morin

**Affiliations:** School of Psychology, Laval University, Quebec, QC G1V 0A6, Canada; marie-christine.ouellet@psy.ulaval.ca (M.-C.O.); cmorin@psy.ulaval.ca (C.M.M.)

**Keywords:** post-traumatic stress disorder, psychological distress, mental health, sleep

## Abstract

This study documents post-traumatic stress symptoms after the May 2016 wildfires in Fort McMurray (Alberta, Canada). A sample of 379 evacuees completed an online questionnaire from July to September 2016, and a subsample of 55 completed a psychiatric/psychological diagnostic interview. According to a self-report questionnaire, 62.5% of respondents had a provisional post-traumatic stress disorder (PTSD). The interview confirmed that 29.1% met criteria for PTSD, 25.5% for depression, and 43.6% for insomnia; in most cases, insomnia was definitely or probably related to the fires. Traumatic exposure may elicit or exacerbate sleep problems, which are closely associated with PTSD after a disaster.

## 1. Introduction

Exposure to a natural disaster is a type of traumatic event that may lead to the development of post-traumatic stress disorder (PTSD). The wildfires that began on 1 May 2016 in Fort McMurray (Alberta, Canada) destroyed 1595 buildings, which contained 2600 housing units, and led to massive displacement of approximately 88,000 people. The fire, which was officially put out in August 2017, after 458 days, devastated a total of 5895 km^2^. Although it caused no direct human fatality, during the evacuation, many individuals faced direct or potential threat to their life or health, or significant losses. Damage was estimated at $3.58 billion, making it the most expensive natural disaster in Canadian history. The inhabitants of the city were displaced at least one month. The city started rebuilding in June 2016, a process expected to take three to four years. Three months after the evacuation, families were still living through ongoing adversity and uncertainty as they adapted to new or temporary homes, schools and workplaces. Beyond the individual experience of the disaster, entire communities have experienced it collectively, facing a variety of challenges at the social, community and economic levels (e.g., housing issues, insurance claims, rebuilding). Both individual and collective stressors are known to contribute to psychological adjustment after a disaster [1].

Specific data on the consequences of major forest fires are not only rare, but their generalizability to other communities may be questioned. Data collected from victims of the 2003 California fires have revealed that two-thirds of the respondents had feared for their lives or the life of a loved one; three months later, a quarter met the criteria for PTSD, and a third met the criteria for major depression [2]. Another study with victims of the Australian bushfires of 1983 demonstrated that health conditions related to stress, including mental disorders, were much more common in this population than conditions unrelated to stress [3]. Three to four years after the Victorian Black Saturday bushfires in Australia (2009), 16–22% of highly affected communities had PTSD and 13% had major depression [4]. To date, two published studies have reported significant issues related to the mental health impacts of the Fort McMurray wildfires. One case-controlled study conducted 18 months after the wildfires showed elevated prevalence of probable self-reported mental health diagnoses (depression, anxiety, alcohol/substance use) in children [5]. The only study reporting mental health indicators in adults was conducted 6 months after the wildfires and focused on self-reported anxiety and alcohol/substance use: Almost 20% of the 486 surveyed participants had a probable diagnosis of generalized anxiety disorder, 14% had high risk of alcohol dependence, and 10% had high risk of drug dependence [6].

Most studies examining the mental health impacts of fires have focused on PTSD, depression, anxiety and substance use [7,8,9]. However, sleep problems—mostly in the form of insomnia and recurrent nightmares, but also sleep-related movement and breathing disorders—are closely associated with both PTSD [10] and depression [11]. Of the 160 studies on the impacts of disasters reviewed by Norris and collaborators (2002), only 10 measured sleep [12]. There is accumulating evidence that persistent sleep problems constitute an important predictor of psychopathology after a traumatic event [13]. Moreover, sleep disturbances appear to be among the most common reactions after a traumatic event: Severe problems with sleep were the most frequently reported complaints two to three weeks after the explosion of a fireworks storage facility in Enschede, The Netherlands, among a vast array of physical and mental health concerns including problems with daily functioning, pain, anxiety and depression [14].

Most people exposed to a disaster experience intense psychological reactions but will remit after some time [15,16]; in parallel, continued stressors contribute to enduring psychological distress, including PTSD [17,18]. Individual factors, such as appraisals of the event and coping strategies, may also influence mental health after a disaster. Ehlers and Clark’s cognitive model suggests that post-traumatic symptoms persist when individuals process information in a way that produces a sense of imminent threat [19]. Two processes are thought to feed this perception of current threat: Recurrent negative appraisals of the trauma and its consequences and the disturbance of autobiographical memory. The latter process is potentiated by unhelpful coping strategies, such as avoidance. Avoidance may concern stimuli that are external (places, people) or internal (memories, thoughts); self-medication with alcohol or other substances may also be a form of avoidance. Sleep difficulties, such as insomnia and nightmares, may play a role as they contribute to physiological arousal and strong emotions, two aspects that may promote the perception of current threat.

In sum, both the wide range of symptoms (e.g., PTSD, anxiety, depression, sleep) and the timeline (e.g., when they occur, how long they persist) are important aspects to consider in the assessment of the mental health impact of a disaster on the affected community. Moreover, due to their unpredictable nature, it is exceptionally difficult to obtain empirical data about the impact of natural disasters. Methodological challenges must be faced: The main bias is often related to the retrospective nature of the data collected, i.e., when we ask people to remember their immediate post-traumatic reactions weeks or even years after the traumatic event [20] and the lack of baseline information on premorbid status. The objective of this research was thus to rapidly document post-traumatic stress symptoms, mental disorders and psychological difficulties in a sample of evacuees of the 2016 Fort McMurray wildfires. The goal was to collect data in the immediate aftermath, i.e., three months after the evacuation, to support self-reported symptoms with standardized clinical interviews, and to assess common mental health disorders (PTSD, depression, anxiety, substance use) as well as sleep problems. We also wanted to examine the associations between symptoms of post-traumatic stress and depression, sleep problems, post-traumatic cognitions and coping strategies. We wished to explore the differential contribution of sleep difficulties in post-traumatic stress severity once the severity of depression was accounted for, as well as the influence of cognitions and coping strategies once the severity of depression and sleep problems were accounted for.

## 2. Method

The Laval University institutional review board approved the research protocol, and participants provided informed consent.

### 2.1. Participants and Procedure

A sample of evacuees from the 2016 Fort McMurray wildfires were asked to complete an online questionnaire. To participate, they had to be aged 18 or older and be fluent in English. A subsample of these volunteers also underwent a standardized clinical interview. The clinical interviews were conducted in the business meeting room of a local hotel.

Two research assistants—doctoral students in clinical psychology—went to Fort McMurray from 25 July to 16 August 2016 to recruit participants and conduct the clinical interviews. They recruited volunteers in various public places (e.g., mall, grocery store). They distributed invitations to complete the online questionnaire, printed on business cards, and also participated in local radio broadcasts to discuss the project and its recruitment. The online questionnaire remained open from 25 July 25 to 5 September 2016. Appointments were taken with individuals interested to participate to the clinical interview, either later in the same day or in the following days. A snowball sampling methodology was also used where participants were invited to refer to the research team other potentially interested acquaintances. The research assistants were easily joinable by phone or email for the whole time of their stay in Fort McMurray.

The research assistants were supervised by the principal investigator (GB), a psychologist specialized in PTSD. They had access to phone supervision as needed for the entire duration of their stay.

### 2.2. Measures

The clinical interview merged two validated standardized diagnostic instruments: The Clinician-Administered PTSD Scale (CAPS) [21] to assess current PTSD, and the Mini International Neuropsychiatric Interview (MINI) [22] to assess current major depression, panic, agoraphobia, generalized anxiety, social phobia and obsessive-compulsive disorders, as well as drug and alcohol use disorders. These instruments provided a stringent examination of the fifth edition of the Diagnostic and Statistical Manual of Mental Disorders (DSM-5) [23] criteria for each disorder. Questions related to the CAPS item #E6 (sleep disturbance) were given a special attention, in order to be able to diagnose insomnia. An insomnia diagnosis was assigned to participants who met the following five criteria: (1) Difficulties falling or staying asleep or waking up too early, and these were (2) present at least three times a week, (3) associated with daytime disturbances, (4) present for at least three months, and (5) occurred despite adequate opportunity for sleep. Questions probing personal antecedents were asked at the end of the interview, i.e., “We just did an overview of the symptoms you have/might have had since the fires. Now I wonder if you have ever experienced problems similar in the past, before the fires. Have you ever had Depression/Insomnia/Recurrent Nightmares/Anxiety Disorder before?”.

The online questionnaires were presented as aimed at studying post-traumatic stress among victims of the Fort McMurray fire. The first questions were open-ended and asked participants to describe their personal experience of the fire and the evacuation, as well as their consequences. Participants then proceeded to complete the PTSD Symptoms Checklist (PCL-5) [24], the Patient Health Questionnaire [25], the Insomnia Severity Index [26], the Pittsburgh Sleep Quality Index and its Addendum for PTSD (PSQI; PSQI-A) [27,28], the Post-Traumatic Cognitions Inventory (PTCI) [29] and the Ways of Coping Questionnaire (WCQ) [30].

The PCL-5 is a 20-item self-report measure that assesses PTSD symptoms in the past month. In this study, we used the PCL-5 to assess symptom severity and to make a provisional PTSD diagnosis. The symptom severity score ranges from 0–80, higher scores indicating more severe symptoms. A provisional PTSD diagnostic may be assigned to respondents who endorse 1 B item (questions 1–5), 1 C item (questions 6–7), 2 D items (questions 8–14), 2 E items (questions 15–20). A cutoff score of 33 is also proposed to discriminate between people with or without probable PTSD [31].

The PHQ is a 59-item questionnaire that was used as a screening tool for mental health disorders of depression, anxiety, alcohol, eating, and somatoform during the past four weeks. From the PHQ, we also used the PHQ-9, a subscale composed of 9 summed items to assess the severity of depressive symptoms [25].

The ISI is a 7-item questionnaire designed to assess the severity of both nighttime and daytime components of insomnia in the past month. Total score ranges from 0–21, higher scores indicating more severe insomnia. A cutoff score of 10 is optimal to detect insomnia in a community sample [32].

The PSQI is an 18-item questionnaire that assesses seven components of sleep quality in the past month: Subjective sleep quality, sleep latency, sleep duration, sleep efficiency, sleep disturbances, use of sleep medication, and impairment of daytime functioning. A global sleep quality score ranging from 0–21 is obtained by summing the seven component scores, higher scores indicating poorer sleep quality. A global sleep quality score higher than 5 is interpreted as poor sleep.

We also used the 7-item addendum to the PSQI, the PSQI-A, to assess the frequency of trauma-related sleep disturbances (hot flashes, general nervousness, memories or nightmares of traumatic experience, severe anxiety or panic not related to traumatic memories, bad dreams not related to traumatic memories, episodes of terror or screaming during sleep without fully awakening and episodes of acting out dreams) in the past month. Total score ranges from 0–21, higher scores indicating more frequent trauma-related sleep disturbances.

The PTCI is a 33-item questionnaire which assessed the degree to which respondents endorsed several post-traumatic cognitions (thoughts) in general on a 1–7 Likert-type scale. Three subscales constitute the PTCI. The Negative cognitions about the self subscale includes 21 items which depict a negative overall opinion of oneself, including a perception of the self as being incapable and weak (e.g., *I am a weak person*), permanently and negatively altered by the event (e.g., *I worsened forever*) and without hope for the future (e.g., *I have no future*). The Negative cognitions about the world/others subscale includes 7 items highlighting the world as a dangerous place and people as inherently unreliable (e.g., *You cannot trust anyone*). The self-blame subscale includes 5 items assessing blame for the occurrence of the traumatic event (e.g., *The event occurred because of the way I acted*). Each subscale score is the average 1-to-7 answer per statement.

Finally, a short version of the WCQ assessed the frequency of use of three coping strategies in the past week: Seeking social support (6 items), positive reappraisal/problem solving (9 items) and distancing/avoidance (6 items). Each item is rated on a 0–3 Likert-type scale. Subscale scores are computed by summing the score of each item, and higher scores indicate more frequent use of the strategy.

### 2.3. Data Analyses

To document post-traumatic stress symptoms, mental disorders and psychological difficulties, frequencies were computed for categorical variables, and means and standard deviations were computed for continuous variables. Lower and upper limits of 95% confidence interval for each mean and for each proportion were computed. Correlations between variables were examined by calculating Pearson’s coefficients. To avoid overlap on the various questionnaires in the correlation and regression analyses, the PCL-5 total score was computed excluding the two sleep items (#2—repeated, disturbing dreams, and #20—trouble falling or staying asleep) and the PHQ-9 total score was computed excluding its sleep item (#3—trouble falling or staying asleep, or sleeping too much). To document the factors associated with the severity of PTSD symptoms, a hierarchical multiple regression analysis was performed, with PTSD symptom severity (PCL-5 total score) as the predicted variable. The observation of the Gaussian distribution of the studentized residuals along with a non-statistically significant Shapiro–Wilk test suggested that the residuals were normally distributed. The first block consisted of gender and age, and was entered with the standard method as a control for the potential effect of these sociodemographic characteristics on PTSD severity. The second block of variables was entered with the standard method and was composed of depressive symptoms (PHQ-9 total score excluding the sleep item). The third block was entered with the standard method and was composed of insomnia severity (ISI total score), global sleep quality (PSQI total score), and trauma-related sleep disturbances (PSQI-A total score). The fourth and final block was entered with the standard method and was composed of negative cognitions about the self (PTCI subscale), negative cognitions about the world (PTCI subscale), self-blame (PTCI subscale), seeking social support (WCQ subscale), problem-solving/positive reappraisal (WCQ subscale) and distancing/avoidance (WCQ subscale). Correlation and regression analyses were performed with SPSS 13.0 for Windows (SPSS Inc., Chicago, IL, USA).

## 3. Results

### 3.1. Sample Description

Three hundred and ninety-four (394) respondents registered to complete the online questionnaire, i.e., went on the website describing the study and clicked to begin the questionnaire. Fifteen questionnaires (3.8%) remained completely or mostly unanswered. Among the 379 completed questionnaires, between 0.02 and 8.4% of data were missing, depending on the question/variable. Missing data were not replaced. Participants were mostly female (77%). Mean age was 40.10 years old (SD = 12.20). Almost three quarters were married or in a common-law relationship (72%); 18% were single, and 7% were either separated, divorced or widowed. Most participants (61%) had children. Residence status in the regional municipality of Wood Buffalo (which includes the city of Fort McMurray) was permanent for 88% and temporary for 7%. Before the fires, most participants worked part or full time (77%); 4% were retired, 3% were on a sick or invalidity leave, and 2% were students. Nearly one participant in four (23%) reported a change in work status since the fires.

Fifty-six (56) respondents participated to the interview. One interview was excluded from the analyses because of incomplete responses: The interviewer suspected that the participant was intoxicated and/or suffering from psychotic symptoms, and redirected the interview to ensure the participant’s safety and access to local mental health resources. The final subsample of 55 participants was practically equally distributed between males (49%) and females (51%). Mean age was 43.07 years old (SD = 14.67). About half were married or in a common-law relationship (49%); 31% were single, and 16% were either separated, divorced or widowed. Most participants (62%) had children. Residence status in the community of Wood Buffalo (which includes the city of Fort McMurray) was permanent for 95% and temporary for 4%. Before the fires, most participants worked part or full time (76%); 4% were retired, 18% were unemployed, and 2% were students. One participant in six (16%) reported a change in work status since the fires.

Although all participants of the clinical interview were invited to complete the online questionnaire, thirty-five (35) did so, representing 9.2% of the sample of completed questionnaires. Compared to respondents who only completed the online questionnaire (344), participants who completed the interview and the online questionnaire (35) were more likely to be male and older, and they showed an overall portrait of less severe mental health problems. They were less likely to have self-reported PTSD, somatoform disorder and major depressive disorder. They showed lower scores on measures of post-traumatic, insomnia and depressive symptoms. They reported less use of avoidance-based coping strategies, less endorsement of cognitions regarding the self, the world or self-blame, and better sleep quality.

### 3.2. Main Results

Data from the online survey showed that, three months after the fires, roughly 60% suffered from significant post-traumatic stress, i.e., they had a provisional PTSD diagnosis according to the PCL-5 or a PCL-5 score of 33 or higher (see Table 1). The most frequently reported symptoms were repeated disturbing memories, feeling upset when reminded of the stressful experience, and trouble falling or staying asleep. Table 2 presents scores obtained on the other self-report questionnaires. The PHQ showed high proportions of respondents endorsing the diagnostic criteria of major depressive disorder (33.1%), somatoform disorder (27.0%) and anxiety disorders other than panic (27.0%). High rates of panic disorder (17.4%), alcohol abuse disorder (17.1%) and binge eating disorder (15.1%) were also observed. Results showed overall high severity of depressive symptoms, insomnia and post-traumatic sleep disturbances, as well as poor sleep quality.

Data from the clinical interviews showed that in this subsample of 55 volunteer participants, 29.1% met the clinical diagnostic criteria for PTSD and 25.5% met the clinical diagnostic criteria for major depression disorder (see Table 3). Among the anxiety disorders, panic disorder and generalized anxiety disorder were the most frequent. Substance use disorders, especially drug use disorder, were diagnosed in 16.7% of the sample. The most frequently encountered diagnosis in this sample was insomnia disorder, with a proportion of 43.6%. Participants were asked whether their sleep difficulties started or got worse after the fires; in most cases, the insomnia disorder was definitely (68%) or probably (16%) related to the fires. Most people with current insomnia (78%) reported having experienced insomnia in the past. PTSD was associated with current insomnia (*Χ^2^*(1) = 17.65, *p* < 0.001), but not with insomnia antecedents (*Χ^2^*(1) = 0.432, *p* = 0.511).

Table 4 presents the associations between the different variables under study. More severe PTSD symptoms was associated with more severe depression and insomnia symptoms, poorer sleep quality and more severe trauma-related sleep disturbances, stronger endorsement of negative cognitions concerning the self, others and self-blame, and with more frequent use of avoidance strategies. PTSD symptom severity was also negatively correlated with age, that is, more severe in younger people.

Results from hierarchical multiple regression analyses indicated that the final model accounted for 77.0% of PTSD symptom severity variance, *F*(8, 340) = 146.722, *p* < 0.001 (Table 5). Sex and age did not contribute significantly to the model (although age was positively associated with symptom severity when no other variable than sex was included in the model). Significant predictors included depressive and insomnia symptoms, trauma-related sleep disturbances, post-traumatic cognitions concerning the others or the world as untrustworthy or dangerous, avoidance-based coping strategies and problem-solving/reappraisal coping strategies. 

## 4. Discussion

The objective of this study was to rapidly document post-traumatic stress symptoms, mental disorders and psychological difficulties in a sample of evacuees in the immediate aftermath of the 2016 Fort McMurray wildfires. Result showed that roughly 60% of respondents to an online questionnaire reported significant post-traumatic stress symptoms, or could be given a provisional PTSD diagnosis according to their responses to a validated self-report questionnaire (PCL-5). This is similar to what was observed among the residents of Enschede, The Netherlands, two or three weeks after the explosion of a fireworks storage facility in a residential area, where more than 50% reported anxious and depressive symptoms of anxiety and sleeping problems, and nearly 75% reported disaster-related reactions of intrusion and avoidance [14]. Four months after a devastating tornado struck the town of Albion, PA in 1985, 76% of its residents also showed high levels of post-traumatic stress [33].

The most frequently reported post-traumatic stress symptoms were repeated disturbing memories (reported by 77.4% of the sample), feeling upset when reminded of the stressful experience (76.7%), and trouble falling or staying asleep (72.5%). The widespread presence of sleep disturbances is a finding consistent with that of studies with larger and more representative samples exposed to a disaster: Rates of severe problems with sleep were threefold that of the general population in the residents of Enschede, The Netherlands, after the fireworks storage facility explosion [14].

Self-report questionnaires suggested alarmingly elevated rates of clinically significant symptoms of major depressive disorder (one in three respondents), but also of somatoform, anxiety and panic disorders, alcohol abuse and binge eating. Respondents reported overall high severity of depressive symptoms, insomnia symptoms and trauma-related sleep disturbances, as well as poor sleep quality. This highlights the relevance to include various outcomes in the assessment of the impact of a disaster on mental health. Because this study did not benefit neither from a comparison with a non-exposed control group nor from an estimation of pre-traumatic mental health of the Fort McMurray population, we cannot directly attribute the observed symptoms to the experience of the fires and of the evacuation. However, all of the observed values were largely greater than prevalence rates of mental health disorders reported in the general population: For example, according to the 2002 Canadian Community Health Survey on Mental Health and Well-Being, the 12-month prevalence of any anxiety disorder is 4.8%, and any mood disorder is 5.3% [34].

We also analyzed data from a subsample of 55 evacuees who participated in a clinical interview where a stringent examination of DSM criteria was conducted. This more rigorous and conservative approach indicated that 29.1% fulfilled the DSM-5 criteria for PTSD. Prevalence of PTSD was somewhat more elevated than that assessed with a DSM-IV-based clinical interview in a sample of individuals three months after an earthquake in North China (18.8%) [35]. However, in the latter study, when researchers calculated the prevalence of PTSD without the requirement for presence of both avoidance and numbing symptoms (DSM-IV’s Criterion C), as is it now the case in DSM-5, they obtained an estimated closer to the one we obtained (27.4%). Almost half of participants with PTSD in the present study (43.6%) could be considered as having an insomnia disorder (recurrently having significant trouble falling or staying asleep). This is about three times higher than in the general Canadian population where the prevalence of insomnia disorder is estimated to be 13.4% [36]. Moreover, although 78% of the subsample reported having had insomnia episodes before the fires, the current insomnia episode was presumably trauma-related, i.e., definitely/probably started or got worse after the fires, in 84% of the cases.

We also wanted to explore the association between of symptoms of post-traumatic stress, and depression, sleep problems, post-traumatic cognitions and coping strategies as assessed three months after the fires. After controlling for sex and age, significant factors associated with post-traumatic stress symptom severity included, depressive symptoms, trauma-related sleep disturbances, post-traumatic cognitions (perceiving other people or the world as untrustworthy or dangerous), insomnia symptoms, avoidance-based coping strategies and problem-solving/reappraisal coping strategies. The observed associations were consistent with the role of cognitions and coping strategies in producing a sense of imminent threat that contribute to maintain and exacerbate post-traumatic stress symptoms [19]. These preliminary findings also suggest that sleep problems, either sleep disturbances or insomnia, are closely associated with PTSD in the aftermath of a disaster, beyond the expected association due to the presence of depressive symptoms. Sleep difficulties may be present before the traumatic event or occur as a result of trauma, and their persistence several days or weeks after the traumatic event may act as a specific risk factor for PTSD [37]. They may constitute symptoms of PTSD, but they may also develop into difficulties concomitant with PTSD, but still different in their nature and their response to treatment [38,39]. Inversely, the treatment of trauma-related sleep problems may have a positive impact on other post-traumatic stress symptoms [40,41]. Interventions to protect or restore sleep after exposure to a disaster are likely to be useful, but the efficacy of sleep management strategies to prevent the development of other mental health problems after traumatic exposure has yet to be studied.

These findings are to be interpreted with caution, as they were assessed very early in the aftermath of the fires and the evacuation, i.e., approximately three months after the evacuation. Some of the respondents had not yet returned home. Furthermore, it is possible that individuals feeling distress could have been more motivated to participate compared to persons with no particular mental health symptoms. Previous findings have shown that resilience, namely the ability to maintain at a stable, healthy level of psychological and physical functioning [15], is to be expected in the majority of individuals. In a study of the aftermath of Hurricane Ike [42], it was found that 75% of the population never developed PTSD when they were evaluated in the 18-month period following the disaster. In addition, between 45–58% did not develop depression, functional impairment, or report days of poor health. Recovery, whereby persons may experience some levels of psychopathology (such as anxiety, depression or sleep problems) but gradually return to pre-event levels of functioning [16], is another common trajectory, where some level of psychological symptoms could be expected as a “normal” reaction to a severely abnormal event, but where symptoms eventually subside. Data collected three to four years after the Victorian Black Saturday bushfires in Australia (February 2009) indicated that more than 80% recovered from symptoms of psychological distress without developing significant mental health problems in the long run [4]. However, studies of resilience and recovery have focused on PTSD and depression, and have not included the assessment of sleep difficulties. Longitudinal follow-ups of the Fort McMurray community will be needed to assess the longer-term evolution of the all of the psychological impacts that were highlighted in these results.

## 5. Conclusions

This study represented a preliminary effort to guide future investigations on the mental health of the Fort McMurray community. A study of the natural history of PTSD, depression and sleep disorders is currently in progress to document the prevalence of psychopathology in the long-term (e.g., three years post-disaster), and to examine the impact of sociodemographic characteristics (e.g., sex/gender, age, ethnicity, income and membership in a First Nation) and degree of loss caused by the fires on prevalence rates. This ongoing study will examine the longitudinal predictors of the course of post-traumatic adaptation, by determining the sociodemographic, psychopathological, cognitive, behavioural and social factors longitudinally associated with not only distress, but also resilience and post-traumatic growth. Finally, we perceived an urgent need in our pool of respondents to be heard about their mental health service needs. Although evidence has shown that patients usually prefer psychological treatment to medication to treat anxiety and depression problems, gender, age and ethnicity greatly influence beliefs and perceived needs in terms of mental health [43]. Data on mental health needs after a disaster from the patients’ perspective are needed to guide the development of large-scale interventions that are accessible and relevant to the affected community.

## Figures and Tables

**Table 1:** Proportion of respondents reporting post-traumatic stress disorder (PTSD) symptoms according to the PTSD symptoms checklist (PCL-5) (*n* = 374–379 ^a^).

Symptom	Number of Respondents Who Endorsed the Symptom (Rated as 2 = “Moderately” or Higher)	Proportion of Respondents Who Endorsed the Symptom (Rated as 2 = “Moderately” or Higher)	95% CI
1. Repeated, disturbing memories (*n* = 376)	291	77.39	[72.90–81.35]
2. Repeated, disturbing dreams (*n* = 374)	203	54.28	[49.21–59.26]
3. Suddenly feeling as if the stressful experience was happening again (*n* = 376)	206	54.79	[49.74–59.75]
4. Feeling very upset when reminded of the stressful experience (*n* = 377)	289	76.66	[72.13–80.65]
5. Strong physical reactions when reminded of the stressful experience (*n* = 377)	250	66.31	[61.40–70.90]
6. Avoidance of memories, thoughts or feelings related to the stressful experience (*n* = 375)	247	65.87	[60.93–70.49]
7. Avoidance of external reminders of the stressful experience (*n* = 376)	226	60.11	[55.08–64.93]
8. Trouble remembering important parts of the stressful experience (*n* = 374)	167	44.65	[39.69–49.72]
9. Strong negative feelings about oneself, other people or the world (*n* = 377)	145	38.46	[33.69–43.46]
10. Blaming oneself or someone else for the stressful experience (*n* = 376)	155	41.22	[36.36–46.26]
11. Strong negative feelings (*n* = 376)	225	59.84	[54.81–64.67]
12. Loss of interest (*n* = 378)	229	60.58	[55.57–65.38]
13. Feeling distant or cut off (*n* = 378)	253	66.93	[62.04–71.48]
14. Trouble experiencing positive feelings (*n* = 378)	197	52.12	[47.09–57.11]
15. Irritable behaviors (*n* = 376)	226	60.11	[55.08–64.93]
16. Taking too many risks (*n* = 377)	72	19.10	[15.45–23.37]
17. Being “superalert” (*n* = 377)	265	70.29	[65.49–74.68]
18. Easily startled (*n* = 378)	229	60.58	[55.57–65.38]
19. Difficulty concentrating (*n* = 378)	262	69.31	[64.49–73.75]
20. Trouble falling or staying asleep (*n* = 378)	274	72.49	[67.78–76.75]
Provisional PTSD diagnosis ^b^ (*n* = 379)	237	62.53	[57.55–67.25]
PCL-5 Total Score 33 or higher (*n* = 379)	226	59.63	[54.62–64.45]

Note: ^a^ On each of the 379 completed PCL-5, between 1 and 5 questions were left unanswered. Missing data were not replaced. ^b^ Proportion of respondents who have endorsed 1 B item (questions 1–5), 1 C item (questions 6–7), 2 D items (questions 8–14), 2 E items (questions 15–20).

**Table 2:** Results on self-report questionnaires (*n* = 347–378 ^a^).

Questionnaire		*n*	%	95% CI
PHQ—Somatoform Disorder (*n* = 378)		102	26.98	[22.75–31.67]
PHQ—Major Depression Disorder (*n* = 378)		125	33.07	[28.52–37.96]
PHQ—Panic Disorder (*n* = 379)		66	17.41	[13.92–21.55]
PHQ—Other Anxiety Disorder (*n* = 378)		102	26.98	[22.75–31.67]
PHQ—Bulimia (*n* = 377)		14	3.71	[2.22–6.13]
PHQ—Binge Eating Disorder (*n* = 377)		57	15.12	[11.86–19.09]
PHQ—Alcohol Abuse (*n* = 375)		64	17.07	[13.06–21.21]
		M	SD	95% CI
Depressive Symptoms (PHQ-9) (*n* = 378)		10.72	6.65	[10.05–11.39]
Insomnia Symptoms (ISI) (*n* = 375)		15.50	7.75	[14.72–16.28]
Trauma-Related Sleep Disturbances (PSQI-A) (*n* = 375)		5.52	4.72	[5.04–6.00]
Global sleep Quality (PSQI) (*n* = 375)		11.76	4.98	[11.26–12.26]
	Sleep Quality (PSQI) (*n* = 375)	1.90	0.85	[1.81–1.99]
	Sleep Latency (PSQI) (*n* = 375)	2.01	1.10	[1.90–2.12]
	Sleep Duration (PSQI) (*n* = 370)	1.76	1.03	[1.66–1.86]
	Sleep Efficiency (PSQI) (*n* = 370)	1.61	1.28	[1.48–1.74]
	Sleep Disturbances (PSQI) (*n* = 375)	1.92	0.76	[1.84–2.00]
	Use of Sleep Medications (PSQI) (*n* = 374)	0.99	1.28	[0.86–1.12]
	Daytime Dysfunction (PSQI) (*n* = 375)	1.62	0.95	[1.52–1.72]
Post-Traumatic Cognitions (PTCI) (*n* = 347)		48.05	43.06	[43.55–52.55]
	Self (PTCI) (*n* = 359)	1.40	1.43	[1.25–1.55]
	World (PTCI) (*n* = 359)	2.25	1.66	[2.08–2.42]
	Self-Blame (PTCI) (*n* = 359)	0.55	0.99	[0.45–0.65]
Seeking Social Support (WCQ) (*n* = 358)		6.70	4.02	[6.28–7.12]
Problem-Solving / Positive Re-Appraisal (WCQ) (*n* = 358)		10.24	6.07	[9.61–10.87]
Distancing / Avoidance (WCQ) (*n* = 359)		6.82	4.15	[6.39–7.25]

Note: ^a^ Among the 379 completed questionnaires, between 1 and 32 data were missing per variable. Missing data were not replaced. M = mean; PHQ = Patient Health Questionnaire; SD = standard deviation.

**Table 3:** Proportions of respondents diagnosed with PTSD or other mental disorders according to clinical interviews ^a^ (*n* = 54 or 55 ^b^).

Diagnosis	Number of Respondents	Proportion of Respondents	95% CI
PTSD (*n* = 55)	16	29.09	[18.77–42.14]
Major Depression Disorder (*n* = 55)	14	25.45	[15.81–38.30]
Panic Disorder (*n* = 55)	13	23.64	[14.37–36.35]
Agoraphobia (*n* = 55)	7	12.73	[6.31–24.02]
Generalized Anxiety Disorder (*n* = 55)	13	23.64	[14.37–36.35]
Social Phobia (*n* = 55)	5	9.09	[3.95–19.58]
Obsessive-Compulsive Disorder (*n* = 55)	0	0.00	--
Drug Use Disorder (*n* = 54)	7	12.96	[6.42–24.42]
Alcohol Use Disorder (*n* = 54)	2	3.70	[1.02–12.53]
Insomnia Disorder (*n* = 55)	24	43.64	[31.38–56.73]

Note: ^a^ PTSD diagnosed with the Clinician-Administered PTSD Scale for DSM-5 (CAPS), Insomnia Disorder diagnosed with questions from the Insomnia Interview Schedule adapted according to the DSM-5 criteria, all other disorders diagnosed with the Mini International Neuropsychiatric Interview (MINI). ^b^ One participant did not provide information to assess alcohol use and another one did not provide information to assess alcohol use.

**Table 4:** Correlation coefficients between variables (*n* = 354–374).

	PTSD	Sex	Age	Depression	Insomnia	Sleep Quality	Sleep Disturbances	Self	World	Self-Blame	Support	Problem-Solving
PTSD												
Sex	0.088											
Age	−0.122 *	−0.177 **										
Depression	0.808 **	0.023	−0.056									
Insomnia	0.673 **	0.082	−0.022	0.726 **								
Sleep Quality	0.564 **	0.107 *	0.044	0.624 **	0.803 **							
Sleep Disturbances	0.702 **	0.118 *	−0.033	0.671 **	0.634 **	0.592 **						
Self	0.730 **	0.028	−0.166 **	0.733 **	0.529 **	0.425 **	0.639 **					
World	0.638 **	−0.042	−0.116 *	0.568 **	0.408 **	0.347 **	0.543 **	0.768 **				
Self-Blame	0.501 **	−0.094	−0.056	0.508 **	0.353 **	0.289 **	0.462 **	0.680 **	0.596 **			
Support	0.053	0.150 **	−0.054	0.100	0.088	0.066	0.115 *	−0.025	0.064	0.043		
Problem-Solving	−0.096	0.012	0.078	−0.090	−0.053	−0.052	0.010	−0.180 **	0.022	−0.014	0.622 **	
Avoidance	0.532 **	0.153 **	−0.248 **	0.464 **	0.414 **	0.351 **	0.444 **	0.505 **	0.445 **	0.351 **	0.196 **	0.134 *

* *p* < 0.05; ** *p* < 0.01; Note: Avoidance: distancing/avoidance (WCQ subscale); Depression: depressive symptoms (PHQ-9 total score excluding the #3 sleep item); Insomnia: insomnia severity (ISI total score); Problem-Solving: problem-solving/positive reappraisal (WCQ subscale); PTSD: post-traumatic symptom severity (PCL-5 total score excluding the #2 and #20 sleep items); Sleep Disturbances: trauma-related sleep disturbances (PSQI-A total score); Sleep Quality: global sleep quality (PSQI total score); Self: negative cognitions about the self (PTCI subscale); Self-Blame: self-blame (PTCI subscale); Support: seeking social support (WCQ subscale), World: negative cognitions about the world (PTCI subscale).

**Table 5:** Multiple regression analyses predicting PTSD symptom severity (excluding sleep items) (*n* = 344).

	Variables	B	SE B	*β*	Adjusted *R*^2^	*R*^2^ Change
Step 1					0.023 **	0.23 **
	Sex	4.778	2.377	0.102		
	Age	−0.176	0.077	−0.123 *		
Step 2					0.680 **	0.654 **
	Sex	2.403	1.363	0.055		
	Age	−0.125	0.044	−0.087 **		
	Depressive symptom severity (PHQ-9 excluding sleep item)	2.439	0.092	0.811 **		
Step 3					0.721 **	0.043 **
	Sex	0.853	1.296	0.019		
	Age	−0.126	0.042	−0.088 **		
	Depressive symptom severity (PHQ-9 excluding sleep item)	1.732	0.138	0.576 **		
	Global sleep quality (PSQI)	−0.141	0.173	−0.040		
	Insomnia symptom severity (ISI)	0.291	0.126	0.128 *		
	Post-traumatic sleep disturbances (PSQI-A)	0.932	0.154	0.249 **		
Step 4					0.753 **	0.035 **
	Sex	1.555	1.263	0.035		
	Age	−0.064	0.041	−0.045		
	Depressive symptom severity (PHQ-9 excluding sleep item)	1.393	0.153	0.465 **		
	Global sleep quality (PSQI)	−0.121	0.164	−0.034		
	Insomnia symptom severity (ISI)	0.287	0.119	0.126 *		
	Post-traumatic sleep disturbances (PSQI-A)	0.646	0.155	0.173 **		
	Negative cognitions about the self (PTCI)	0.227	0.736	0.018		
	Negative cognitions about the world/others (PTCI)	1.827	0.463	0.172 **		
	Self-blame (PTCI)	0.046	0.681	0.002		
	Seeking social support (WCQ)	−0.166	0.157	−0.038		
	Positive reappraisal/Problem solving (WCQ)	−0.107	0.107	−0.037		
	Distancing/Avoidance (WCQ)	0.461	0.145	0.109 **		

* *p* < 0.05; ** *p* < 0.01. B = beta weight; SE B = standard error of the beta weight.
